# The Escalating Magnitude of COVID-19 Infections among the Northeastern Ethiopia Region: A Community-Based Cross-Sectional Study

**DOI:** 10.1155/2021/5549893

**Published:** 2021-05-04

**Authors:** Zeleke Geto, Saba Gebremichael, Melaku Ashagrie Belete, Alemu Gedefie, Genet Molla, Melkam Tesfaye, Wondmagegn Demsiss, Daniel Gebretsadik

**Affiliations:** ^1^Department of Biomedical Science, College of Medicine and Health Science, Wollo University, Wollo, Dessie, Ethiopia; ^2^Department of Medical Laboratory Science, College of Medicine and Health Science, Wollo University, Wollo, Dessie, Ethiopia

## Abstract

**Background:**

The novel coronavirus disease (COVID-19) is lethal and extremely contagious, with a rapidly rising global prevalence. The World Health Organization has declared the outbreak a global pandemic; it is reported to have spread to nearly every country in the world. However, the prevalence varies across developed and developing countries, as well as within different regions of the same country. It is not hidden that estimating the magnitude of COVID-19 infection from the community surveys is critical for public health policymakers to make decisions to deal with the outbreak, optimize measures, and design mitigation plans.

**Methods:**

A community-based cross-sectional study was conducted from 01 July to 31 August 2020 in the northeastern Ethiopia region. A simple random sampling technique was used to select study participants from the community survey, contact traces from confirmed cases, and infection suspects. After extraction of viral nucleic acid from oropharyngeal specimen, the real-time fluorescent polymerase chain reaction (RT-PCR) kit was used for detecting novel coronavirus.

**Results:**

A total of 8752 study participants were included in this study. About 63.6% were males and 36.4% were females. Out of the total 8752 study participants, 291 (3.3%) were found to be infected with the virus. The first laboratory-confirmed cases of COVID-19 were detected in the fourth week of the study period, that is, from July 24 to July 31, 2020, and the peak prevalence was observed in the last two weeks. The COVID-19 infection was more prevalent among males and in the age group of 36–52 years. Participants tested via contact trace had 1.65 times (AOR = 1.65, 95% CI = 1.09–2.51, *P*=0.018) the likelihood of COVID-19 infection in comparison to the other forms of community surveys.

**Conclusion:**

The trend in the prevalence of COVID-19 infection in the northeastern region has shown increment, and increasing testing capacity has a greater benefit in identifying early infection for the prevention, treatment, and control of the international pandemic.

## 1. Introduction

The novel coronavirus disease (currently known as COVID-19) is a lethal and extremely contagious respiratory illness caused by the novel coronavirus, now named as severe acute respiratory syndrome coronavirus 2 (SARS-CoV-2) [1–3]. It has become the current global health threat and an international public health emergency [4]. The novel coronavirus was first detected in late December 2019 and believed to be originated from bats, snakes, and different raw sea foods in Wuhan, Hubei Province of China [5, 6]. The World Health Organization (WHO) declared COVID-19 as a pandemic disease on March 11, 2020 [7]. Currently, COVID-19 is reported to have expanded to almost all countries of the world, raising great public health concerns globally, and since the start of the outbreak, the global pandemic surpasses 124 million cases worldwide and the death toll is more than 2.7 million [8]. Since the first COVID-19 case in Africa was recorded on February 14, 2020, in Egypt, followed by Algeria on February 25, 2020 [9], there have been more than 2.9 million reported confirmed cases and more than 75,000 deaths across the continent as of March 19, 2021 [10]. This is a large total, but it is lower than the high figures projected [11, 12] at the onset of the pandemic. Despite having 17% of the global population, Africa has only accounted for 5% of global COVID-19 confirmed cases and 3% of global COVID-19 deaths [13–15]. COVID-19 cases continued to be reported along the southern and eastern regions of Africa with varying numbers mainly due to economic vulnerability and inadequate access to personal protective equipment [16–18]. So far, a lesser number of cases have been reported from African countries, which is debatable. Some of the speculations are lower testing rates, poor reporting habits, and lower number of passengers coming to the continent from high burden countries [12, 13, 19–21].

In Ethiopia, the first case of COVID-19 was reported on 13th March 2020 [22]. Currently, the infection rate is rapidly increasing, with more than 190,000 total cases and over 2693 deaths were reported in Ethiopia [10]. This might look as a small figure when compared with other countries; however, this figure was reported from a total of 1.2 million total numbers of tests. In developing countries such as Ethiopia, primarily due to insufficient testing capacity, the total number of cases might be underestimated and expected to be more than what is being reported. As a result, by boosting the testing capacity in different parts of the country, the exact current figure needs to be known, and the distribution among various regions of the country needs to be identified. For such reasons, community-based, massive COVID-19 testing services and health mobilization are necessary in the country. The national COVID-19 prevalence is not clearly known and can vary from region to region. However, public health policymakers need to know the magnitude of infection in order to make decisions about how to deal with the outbreak, optimize measures, and design the mitigation plans for prevention and control. Having this, we aimed to estimate the magnitude of COVID-19 infection in the country's northeastern region using coronavirus community test data from Wollo University's COVID-19 testing laboratory.

## 2. Methods

### 2.1. Study Design, Area, and Period

A community-based cross-sectional study was conducted from 01 July to 31 August 2020 in northeastern Ethiopia. The study area included three zones: South Wollo Zone, Afar Regional State (nearest to the northeast region), and Oromia Special Zone in northeastern Ethiopia. There are two COVID-19 testing centers in this region of the country: Wollo University COVID-19 Laboratory Testing Center and Amhara Public Health Institute (APHI), Dessie branch. Moreover, each woreda and city has COVID-19 screening, sample collection, quarantine, and isolation centers. The catchment population for the geographical locations involved in this study is estimated to be more than 10 million people.

### 2.2. Study Populations

The source population comprises of all communities in the northeastern Ethiopia region. The study participants were selected using a simple random sampling technique from those who were suspected of having COVID-19, had contact with known COVID-19 cases, had symptoms of acute respiratory illnesses, and who were apparently healthy individuals involved as part of community surveillance in the northeastern Ethiopia region during the study period.

### 2.3. Data and Specimen Collection

WHO standardized check list was used to obtain information of the study participants related to sociodemographic characteristics, clinical, and risk factor data [23]. Oropharyngeal specimen was collected using a sterile cotton swab and placed (dipped) immediately into prelabeled (date, time, and identification code), leak-proof, sterile, screw-capped plastic transport tube containing 2-3 mL viral transport medium (VTM) by a trained healthcare provider following proper infection control strategies and using recommended personal protective equipment (PPE). All specimens collected for laboratory investigations were regarded as potentially infectious, and appropriate precautionary safety procedures and standard operating procedures (SOPs) were maintained throughout specimen collection and handling.

### 2.4. Specimen Packaging, Storage, and Transportation

The collected specimens were then placed in the tube rack and stored in a cold box which maintains 2–8°C maintaining the triple packaging procedure. The properly packaged oropharyngeal specimens were then transported to Wollo University COVID-19 Laboratory from the collection sites. When there is likely to be an unavoidable delay in specimens being tested in the laboratory, the specimens were kept refrigerated at 2–8°C until being processed.

### 2.5. Extraction of Viral Nucleic Acid

About 200 *μ*L of the collected oropharyngeal specimen was mixed with the prepared lysis working solution and proteinase K in 1.5 mL sterile centrifuge tube to quickly dissolve the protein and make nucleic acid dissociated. Then, the dissociated nucleic acid compositions were combined with the silicone membrane of spin column after the addition of ethyl alcohol. Inhibitor remover and deionized solution were added to remove the protein, inorganic salt ions, and many organic impurities in a cascade of washing and centrifugation steps, and then, eluent was added to elute pure nucleic acid. The final pure nucleic acid solution was immediately processed for viral detection, and the rest was preserved by storing at −20°C, incase repeating the test would be necessary [24].

### 2.6. Detection and Amplification

The real-time fluorescent polymerase chain reaction (RT-PCR) kit was used for detecting the novel coronavirus (COVID-19). It is based on a qualitative in vitro nucleic acid amplification assay intended to detect ORF1ab gene of COVID-19 using reverse transcription PCR combining fluorescent probing. Primers and sequence-specific fluorescence probes were designed tailored to the high conservative region in COVID-19 genome. The probes were oligonucleotide attached fluorophores at the 5′ end with FAM as reporter and 3′ end with quencher. In the meantime, specific primers and probes were developed as internal reference with the fluorophore VIC attached at the 5′ end as reporter. Immediately after being out from the −20°C storage, the kit contents were thawed thoroughly at ambient temperature except the enzyme mix. About 20 *μ*L PCR-mixed ingredients (master mix) were prepared and mixed with 10 *μ*L of previously extracted nucleic acid solution in 96-well PCR plates, the plate was then loaded into the RT-PCR machine, and an automatic process completes the remaining assay steps. Monitoring the fluorescence intensity, shape of FAM and VIC fluorophores, and cycle threshold values (Ct values) during the automated RT-PCR allowed the qualitative detection of COVID-19 as positive or negative [25–27].

### 2.7. Quality Assurance

To generate quality and reliable data, all quality control checks were performed in the whole data collection process. Specimen collection, transportation protocols, and special safety precautions were provided with the necessary job aids for the facilities under study to monitor their process quality. Moreover, all laboratory assays were performed by maintaining quality control procedures. Standard operating procedures (SOPs) were strictly followed through all aspects of data collection including specimen collection, handling, testing, and infection control strategies. No template control (NTC), which was composed of nuclease-free water, was included in each run to monitor reagent and system contamination during the RNA extraction process.

A negative control and positive control were used for every run to verify that sample processing, amplification, and detection steps were performed correctly. Viral RNA genome extraction was performed in a level 2 biosafety cabinet (BSL-2). To remove contamination, the sample preparation (BSC II, type A2) and amplification instruments were cleaned regularly based on the decontamination and cleaning protocol found within the operator manual. The data from the standardized check list were checked for its completeness and accuracy. And data cleaning and double data entry were applied to assure quality of the data.

### 2.8. Statistical Analysis

Data were entered to Microsoft Excel and exported to SPSS version 22 software (IBM, USA) and analyzed. Descriptive statistics were summarized in tables and graphs. The chi-square test for the association between dependent and independent variables was used. Binary logistic regression was employed to show the association of each variable with the dependent variable. Moreover, a multivariate analysis was computed to identify factors that independently influence the occurrence of the dependent variable. The level of significance was set to 0.05 (*α* = 5%) with a corresponding 95% confidence interval.

### 2.9. Ethical Considerations

Ethical clearance was obtained from the Department of Medical Laboratory Science, College of Medicine and Health Sciences, Wollo University. Moreover, informed consent was obtained from each study participant or from parents/guardians for participants who could not give consent by themselves. The objectives of the study were explained to the participants by the data collectors. The study was conducted according to the principles expressed in the Declaration of Helsinki. Study participants were aware regarding the threats posed by the pandemic and the significance of being tested. Participants were also informed that positive results were communicated with the zonal and regional health offices, so that further contact tracing of positive individuals as well as proper management of positive individuals including quarantine, isolation, or critical patient care has been performed.

## 3. Results

### 3.1. Sociodemographic Characteristics

About 8752 laboratory-tested individuals were included in this study between 01 July 2020 and 31 August 2020. The mean (±SD) age of the study participants was 31.6 (±13.6). Sixty percent (60%) of the study participants were between the age group of 36 and 52 years. The sex ratio was skewed towards males. More than fifty percent (52.2%) of the individuals were permanently living in South Wollo Zone, and almost all study participants were from different parts of Amhara regional state (Table 1).

### 3.2. Prevalence of COVID-19 Infections among Study Participants

The number of laboratory-confirmed COVID-19 infections was higher in males and in the age group of 36–52 years, but the infection was more prevalent among individuals who were in the age category of 87–104 years (5%). Thirty-three of the study participants had a travel history to the neighboring country, Djibouti, but all of them were negative for COVID-19 infection. Out of the total COVID-19 screened participants, the majority of positive cases were from the community surveillance which accounts 2.2%. About 233/291 COVID-19 infections were detected in the final quarter of the study period, accounting for the majority (2.7%) of the total 3.3% infection rate (Table 2).

### 3.3. Trends of COVID-19 Infection among Study Participants

**Figure 1:**
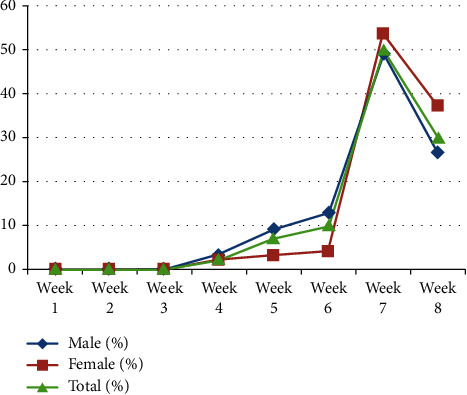
Trends of COVID-19 infection in the northeastern region of Ethiopia from July 01 to August 31, 2020.

COVID-19 infections had started increasing in prevalence in the fourth week of the study period, from July 24 to July 31, 2020, and peak prevalence was observed in the last two weeks. No positive cases were found during the first three weeks of the massive testing period of COVID-19 infection. From week 4 to week 6, however, the positivity rate of the infection was higher among males than female study participants. Female study participants, on the other hand, had a higher prevalence of COVID-19 infection in the last two weeks than males (Figure 1).

As given in Table 3, the trends of COVID-19 testing generally increased along all weeks and age groups of the mass screening period, with the highest number of tests performed at week 7. The number of people infected with COVID-19 infection has increased proportionally as testing capacity has increased. At week 7, a greater number of tests were conducted and a higher number of individuals infected with COVID-19 infection were identified. The trend in the number of individuals tested for COVID-19 infection was also increased across each zone. Positive cases were identified among study participants and were further regularly screened at follow-up centers. Follow-up testing was started in week six, whereas mass screening was started in week five (August 01, 2020).

### 3.4. COVID-19 Infection and Associated Factors among Study Participants

Except for the gender parameter, the rest of the variables in the current study were found to have a statistically significant association with the laboratory test result of COVID-19 screening. Participants aged 87–104 years were 6 times (AOR = 6.03, 95% CI = 1.19–30.4, *P*=0.03) more likely to be infected with COVID-19 than those aged 1–35 years. Living in South Wollo Zone (AOR = 0.56, 95% CI = 0.39–0.79, *P*=0.001) and Wag Hemra (AOR = 0.59, 95% CI = 0.36–0.99, *P*=0.044) as permanent inhabitant had shown an inverse association with COVID-19 infection in multivariate logistic regression analysis. Participants who were tested via contact trace had 1.65 times (AOR = 1.65, 95% CI = 1.09–2.51, *P*=0.018) the likelihood of COVID-19 infection in comparison to the other forms of community survey (Table 4).

## 4. Discussion

In the present community-based cross-sectional study conducted from 01 July to 31 August 2020, a total of 8752 study participants were involved. Out of the total study participants, 291 (3.3%) were found to be infected with the virus with an overall prevalence of 3.3%, which is in line with a facility-based study conducted in Ataye, Northeast Ethiopia (3.3%) [28]. However, this prevalence is higher than population survey reports from Iceland (0.6%) [29], Luxembourg (0.3%) [30], and Slovenia (0.15%) [31]. On the contrary, the prevalence of COVID-19 in the current study was lower than a study conducted in Pakistan (14.3%) [32] and Djibouti (8%) [33] and a population survey report in Sudan (42.9%) [34]. This difference in prevalence might be due to variation in population, study period, testing ability, and study subjects.

In the current study, the rate of infection showed increment in the course of time. There were no any laboratory-confirmed positive cases during the first 15 days of the study period (July 1–15, 2020), and then, it reached 1.1%, 2.1%, and 4.3% in the second, third, and fourth 15 days, respectively. This finding is not in agreement with a population screening report in Iceland where the percentage of infected participants remained steady during the screening period in the country [29]. The increment in the incidence of infection over time might be due to a reduction in the community's habit of sticking to the containment precautions imposed by the Ethiopian health authorities. As it is well known, Ethiopia is among low-income countries, as a result of which the population may not fully practice the measurements needed to combat the pandemic like home isolation, self-quarantine, and other social distancing measures that may have helped to prevent an increase in the rate of infection. Another possible justification for an increase in the rate of infection might be the national surveillance campaign of COVID-19 during the study period. This, in turn, showed that the virus is already spread and disseminated in the community.

A study performed in Pakistan reported that male study participants, 85 (70.25%), were more affected by the pandemic than female counterparts and 36 (29.8%) with an observed significant difference (*P* < 0.001) [32]. On the contrary, a study conducted in China indicated that females had a higher rate of confirmed cases compared with their counterparts, but males were more likely to have severe or critical illness [35]. Eventhough the difference is not statistically significant, the positivity rate of COVID-19 infection in the current study is slightly higher among males than females (3.5% vs. 3%). There are a number of studies regarding the possible reasons for the skewed rate of positivity of COVID-19 between male and female study participants. A study that has been conducted in Ethiopia indicated that males were more likely to be more knowledgeable than their female counterparts [36]. A recent study in the northeastern Ethiopia region has also revealed that being female is significantly associated with low level knowledge about the transmission, prevention, and control of COVID-19 pandemic. According to this study, female study participants had 32 times odds of having low level of knowledge in comparison with their male counterparts (AOR = 32, 95% CI: 7.66–133.7, *P* < 0.001) [28]. Receiving health information related to COVID-19 by the population may render more frequent efforts to engage in all kinds of preventive behaviors, such as wearing a facemask in public, washing hands, and so on [37]. On the other hand, a few studies conducted elsewhere [37–39] reported that male study participants were likely to have low level of practice towards the control and prevention of the pandemic. A similar study in Cameroon revealed that women had lower practice scores compared to men [40]. However, other studies did not reveal a statistically significant association between gender and the level of practice of the study participants towards COVID-19 prevention and control measures [28, 41].

The prevalence of COVID-19 was increasing across age categories in the present study. Its prevalence among study participants in the age category of 1–35 years, 36–52 years, 53–69 years, 70–86 years, and 87–104 years was 27 (2.7%), 174 (3.3%), 60 (3.2%), 22 (3.8%), and 8 (5.0%), respectively. Similarly, elderly study participants were found to be more affected by COVID-19, and the positivity rate was found to be higher [32]. This increasing positivity rate across the age group might be attributed to different variables such as knowledge, practice, and possible exposure to numerous chronic diseases. Besides, several studies have indicated that older age is one of the most important determinants for the occurrence of chronic disease [42–44]. Most of the fatal cases and severe illnesses due to COVID-19 occurred in elderly individuals and people who have underlying medical conditions such as diabetes, cancer, hypertension, and heart, lung, and kidney diseases [45, 46].

Of the total study participants, 33 (0.38%) had a history of travel from Djibouti, but none of them were found to be confirmed (positive) cases of COVID-19. The first confirmed case in Djibouti was reported on 18 March 2020; the total number of confirmed cases in the country as of 18 April 2020 was 732, while it was only 96 in Ethiopia [12]. Since the start of COVID-19 pandemic, mainly due to geographical proximity, many people including Ethiopian long-distance vehicle drivers, traders, and others were migrating from Djibouti to Ethiopia via the route that has connection with the Amhara region, particularly South Wollo Zone and Oromia Special Zone. This lower reported rate of COVID-19 from study participants who had a travel history to and from Djibouti might indicate partly the tendency of study participants to hide their travel history due to fear of community stigma [47] and, in part, due to their good practice of COVID-19 control and prevention measures. In other ways, the recent travel history to high-risk countries, rather than any simple travel history, might be linked to the potential high risk of transmission of COVID-19 [29].

The number of COVID-19 confirmed cases among newly diagnosed individuals, 256 (2.9%), was higher than those study participants who were under follow-up, 35 (0.4%). This might indicate a bad impression that the virus is already disseminated in the community of our testing area and might also indicate a good recovery rate of the known positive cases. A similar scenario took place in a study conducted in Djibouti where very few numbers of primary cases were able to disseminate the virus to large number of the community members [33]. In contrast to the current study, a low recovery rate was reported in China, where 41.8% of the study participants developed acute respiratory distress syndrome and 21.9% of the total COVID-19 positive cases died [48].

The four catchment areas investigated in the current study were Dessie town, South Wollo Zone, Oromia Special Zone, and Wag Hemra Zone. Study participants whose permanent residence was outside these catchment areas and who provided specimen in any of the four areas were considered to have an intercity travel history. Accordingly, in relation to study participants who had permanent residency in Dessie town, the likelihood of acquiring COVID-19 infection was more than 2 times higher among study participants who had an intercity travel history (AOR = 2.03, 95% CI = 1.03–3.99, *P*=0.041). These study participants might come from areas (cities) where more COVID-19 cases were reported, such as Addis Ababa, Gondar, and Gojam, indicating that there was no travel restriction within the country. A study in China indicated the impact of travel restrictions to delay the spread of the pandemic from Wuhan, where COVID-19 was started, to mainland China [49].

This surveillance was conducted to check the dissemination pattern of the pandemic in the community among suspected cases, among those who had a contact history, among those working in different facilities, and among follow-up cases. In the current study, individuals who have been suspected by the physician of developing COVID-19 infection did not have a statistically significant association with the positivity rate. Due to the similarity of signs and symptoms of COVID-19 with pneumonia and other types of respiratory diseases, physicians (clinicians) could suspect individuals with other morbidities as COVID-19 infection and send their specimen for laboratory confirmation. In the present study, having a history of contact with confirmed cases of COVID-19 had 1.65 times higher odds of acquiring the infection. Quarantine of patients and close contact appear to have been associated with a reduction in COVID-19 transmission in China. On the contrary, if contact of the primary cases with noninfected individuals could not be controlled, the transmission of infection would be paramount. This fact can be supported by a study in Djibouti where the rate of primary cases was only 2%, whereas the rate of infection among study participants who had a history of contact was substantial [33].

## 5. Conclusion

The trend in the prevalence of COVID-19 infection in the northeastern region has shown a rise in tandem with the expansion of testing capacity. The prevalence of the virus was found to be significantly higher among old-aged individuals, intercity travelers, and those who had contact with confirmed/clinical suspects of COVID-19. Whereas, its prevalence is significantly low in relatively semiurban or less dense areas of the survey. Although Ethiopia has taken some steps to detect, manage, and control the transmission of COVID-19, more effort is needed to expand testing capacity and bring about behavioral changes in the community in order to halt spread of the pandemic. The country needs to put in place alternative options to mitigate interruptions of essential healthcare services and scientific research studies of significant impact.

### 5.1. Limitations

This study employed a cross-sectional study design, which could not draw conclusions regarding causality and alternative explanations for the findings. Clinical and risk factor data were not included in the study. Moreover, the present study did not address the molecular level sequence of the viral nucleic acid from the confirmed cases nor did it include data on hematological and clinical chemistry parameters.

## Figures and Tables

**Table 1:** Sociodemographic characteristics of the study participants in the northeastern region of Ethiopia, 2020.

Category	Individuals (8752)
Age, years	
Mean (SD)	31.6 (13.6)
Range	1–104

Age groups, *n* (%)	
1–18	30 (0.4)
19–35	862 (9.8)
36–52	5236 (59.8)
53–69	1880 (21.5)
70–86	585 (6.7)
87–104	159 (1.9)

Sex, *n* (%)	
Male	5568 (63.6)
Female	3184 (36.4)

Ethnic origin	
Ethiopia	8747 (99.9)
Others (China, Oman, and Taiwan)	5 (0.1)

Permanent residence (region)	
Amhara	8728 (99.7)
Afar	19 (0.2)
Others (Addis Ababa, Benishangul-Gumuz)	5 (0.1)

Permanent residence (zone)	
South Wollo	4572 (52.2)
Dessie town	1175 (13.4)
Oromia Special Zone	1893 (21.6)
Wag Hemra	980 (11.2)
Others (North Wollo, Showa, Gojam, Gondar, and Afar)	132 (1.5)

**Table 2:** Prevalence of COVID-19 infection among study participants in the northeastern region of Ethiopia, 2020.

	*n* (%) of total	Positive	Negative	*P* value
Sex				
Female	97 (1.1)	97(3.0)	3087 (97.0)	0.272
Male	194 (2.2)	194 (3.5)	5374 (96.5)	

Age, years				
1–35	27 (0.31)	27 (2.7)	965 (97.3)	0.053
36–52	174 (2.0)	174 (3.3)	5062 (96.7)	
53–69	60 (0.7)	60 (3.2)	1820 (96.8)	
70–86	22 (0.3)	22 (3.8)	563 (96.2)	
87–104	8 (0.1)	8 (5.0)	151 (95)	

Time period of screening				
July 1–July 15, 2020	0 (0)	0 (0)	179 (100)	<0.005^*∗*^
July 16–July 31, 2020	8 (0.1)	8(1.1)	717 (98.9)	
August 1–August 15, 2020	50 (0.6)	50 (2.1)	2352(97.9)	
August 16–August 31, 2020	233 (2.7)	233(4.3)	5213(95.7)	

Travel history				
Yes (Djibouti)	33 (0.4)	0	33 (100)	0.286
No	291 (3.3)	291 (3.3)	8428 (96.7)	

Individual classification for testing				
New	256 (2.9)	256 (3.0)	8310 (97.0)	<0.005^*∗*^
Follow-up	35 (0.4)	35 (18.8)	151 (81.2)	

Reason for testing (newly tested and follow-up)				
Community surveillance	189 (2.2)	189 (2.7)	6808 (97.3)	<0.005
Suspect	37 (0.4)	37 (3.9)	919 (96.1)	
Contact	28 (0.3)	28 (5.1)	516 (94.9)	
Facility-based survey	2 (0.1)	2 (2.9)	67 (97.1)	
Follow-up	35 (0.4)	35 (18.8)	151 (81.2)	

**Table 3:** Trend of COVID-19 infection in the northeastern region of Ethiopia from July 01, 2020, to August 31, 2020, by different variables across each week (*N* = 291).

Parameters		Week 1	Week 2	Week 3	Week 4	Week 5	Week 6	Week 7	Week 8
Age, years									
1–35	Negative	48 (0.8)	54 (0.9)	186 (3.1)	386 (6.5)	521 (8.8)	1317 (22.2)	2508 (42.3)	907 (15.3)
	Positive	0 (0)	0 (0)	0 (0)	6 (3.0)	17 (8.5)	20 (10)	104 (51.7)	54 (26.9)
36–52	Negative	14 (0.7)	14 (0.7)	21 (1.1)	124 (6.8)	108 (5.9)	330 (18.1)	819 (45.0)	390 (21.4)
	Positive	0 (0)	0 (0)	0 (0)	1 (1.7)	3 (5.0)	5 (8.3)	29 (48.3)	22 (36.7)
53–69	Negative	6 (1.0)	9 (1.5)	2 (0.3)	25 (4.4)	29 (5.0)	79 (14.0)	243 (43.2)	170 (30.2)
	Positive	0 (0)	0 (0)	0 (0)	1 (4.5)	0 (0)	3 (13.6)	9 (40.9)	9 (40.9)
70–86	Negative	2 (1.3)	4 (2.7)	0 (0)	1 (0.7)	8 (5.4)	28 (19.6)	52 (36.4)	48 (33.6)
	Positive	0 (0)	0 (0)	0 (0)	0 (0)	0 (0)	1 (16.7)	4 (66.7)	1 (16.7)
87–104	Negative	0 (0)	0 (0)	0 (0)	0 (0)	1 (12.5)	2 (25.0)	5 (62.5)	0 (0)
	Positive	0 (0)	0 (0)	0 (0)	0 (0)	1 (50.0)	0 (0)	0 (0)	1 (50.0)

Permanent residence by zone									
Dessie town	Negative	0 (0)	0 (0)	0 (0)	0 (0)	0 (0)	28 (2.5)	527 (46.9)	668 (50.6)
	Positive	0 (0)	0 (0)	0 (0)	0 (0)	0 (0)	3 (5.8)	9 (17.3)	40 (76.9)
South Wollo	Negative	47 (1.0)	42 (0.9)	189 (4.2)	352 (7.9)	487 (10.9)	1147 (25.7)	1723 (38.6)	473 (10.6)
	Positive	0 (0)	0 (0)	0 (0)	2 (1.8)	15 (13.4)	8 (7.1)	73 (65.2)	14 (12.5)
Oromia Zone	Negative	5 (0.3)	10 (0.6)	20 (1.1)	88 (4.9)	119 (6.6)	201 (10.5)	947 (52.6)	422 (23.4)
	Positive	0 (0)	0 (0)	0 (0)	3 (3.3)	6 (6.5)	11 (12.0)	42 (45.7)	30 (32.6)
Wag Hemra	Negative	18 (1.8)	29 (3.0)	0 (0.0)	83 (8.7)	61 (6.2)	388 (40.6)	373 (39.0)	4 (0.4)
	Positive	0 (0)	0 (0)	0 (0)	1 (4.2)	0 (0)	7 (29.2)	16 (66.7)	0 (0)
Others	Negative	0 (0)	0 (0)	0 (0)	13 (10.7)	0 (0)	3 (2.5)	57 (47.1)	48 (39.7)
	Positive	0 (0)	0 (0)	0 (0)	2 (18.2)	0 (0)	0 (0)	6 (54.5)	3 (27.3)

Classification of study subjects for COVID-19 screening									
New	Negative	70 (0.8)	80 (1.0)	209 (2.5)	536 (6.5)	667 (8.0)	1708 (20.6)	3564 (42.9)	1476 (17.8)
	Positive	0 (0)	0 (0)	0 (0)	8 (3.1)	21 (8.2)	28 (10.9)	132 (51.6)	67 (26.2)
Follow-up	Negative	0 (0)	1 (0.7)	0 (0)	0 (0)	0 (0)	48 (31.8)	63 (41.7)	39 (25.8)
	Positive	0 (0)	0 (0)	0 (0)	0 (0)	0 (0)	1 (2.9)	14 (40.0)	20 (57.1)

Reason for testing									
Community surveillance	Negative	10 (0.1)	25 (0.4)	63 (0.9)	394 (5.8)	546 (8.0)	1618 (23.8)	3182 (46.7)	970 (14.2)
	Positive	0 (0)	0 (0)	0 (0)	6 (3.2)	9 (4.8)	19 (10.1)	108 (57.1)	47 (24.9)
Suspect	Negative	24 (2.5)	41 (4.3)	146 (15.3)	29 (3.0)	61 (6.6)	28 (3.0)	275 (29.9)	315 (34.3)
	Positive	0 (0)	0 (0)	0 (0)	0 (0)	6 (16.2)	9 (24.3)	14 (37.8)	8 (21.6)
Contact	Negative	36 (7.0)	14 (2.7)	0 (0)	46 (8.9)	60 (11.6)	62 (12.0)	107 (20.7)	191 (37.0)
	Positive	0 (0)	0 (0)	0 (0)	0 (0)	6 (21.4)	0 (0)	10 (35.7)	12 (42.9)
Facility-based survey	Negative	0 (0)	0 (0)	0 (0)	69 (100)	0 (0)	0 (0)	0 (0)	0 (0)
	Positive	0 (0)	0 (0)	0 (0)	2 (100)	0 (0)	0 (0)	0 (0)	0 (0)
Follow-up	Negative	0 (0)	1 (0.7)	0 (0)	0 (0)	0 (0)	49 (31.8)	63 (41.7)	39 (25.8)
	Positive	0 (0)	0 (0)	0 (0)	0 (0)	0 (0)	1 (2.9)	14 (40.0)	20 (57.1)

**Table 4:** COVID-19 infections and associated factors among study participants in the northeastern region of Ethiopia, 2020.

	Positive	Negative	COR (95% CI)	*P* value	AOR (95% CI)	*P* value
Sex						
Female	97 (3.0)	3087 (97.0)	1.00		1.00	
Male	194 (3.5)	5374 (96.5)	1.15 (0.9–1.47)	0.272	1.17 (0.91–1.50)	0.230

Age, years						
1–35	27 (2.7)	965 (97.3)	1.00		1.00	
36–52	174 (3.3)	5062 (96.7)	0.14 (0.03–0.64)	0.012	1.02 (0.76–1.38)	0.871
53–69	60 (3.2)	1820 (96.8)	0.13 (0.03–0.63)	0.011	1.19 (0.75–1.87)	0.466
70–86	22 (3.8)	563 (96.2)	0.16 (0.03–0.78)	0.024	1.15 (0.49–2.67)	0.749
87–104	8 (5.0)	151 (95)	0.17 (0.03–0.97)	0.046	6.03 (1.19–30.4)	0.030^*∗*^

Permanent residence by zone						
Dessie town	52 (4.4)	1123 (95.6)	1.00		1.00	
South Wollo	112 (2.4)	4460 (97.6)	0.54 (0.39–0.76)	<0.005	0.56 (0.39–0.79)	0.001^*∗*^
Special zone of Oromia	92 (4.9)	1801 (95.1)	1.10 (0.779–1.56)	0.580	0.98 (0.68–1.41)	0.92
Wag Hemra	24 (2.4)	956 (97.6)	0.54 (0.33–0.87)	0.015	0.59 (0.36–0.99)	0.044^*∗*^
Others	11 (8.3)	121 (91.7)	1.96 (0.998–3.86)	0.051	2.03 (1.03–3.99)	0.041^*∗*^

Classification of study subjects for COVID-19 screening						
New	256 (3.0)	8310 (97.0)	7.52 (5.1–11.1)	<0.005	7.63 (5.07–11.5)	<0.005^*∗*^
Follow-up	35 (18.8)	151 (81.2)	1.00		1.00	

Reason for testing						
Community surveillance	189 (2.7)	6808 (97.3)	1.00		1.00	
Suspect	17 (3.9)	919 (96.1)	1.45 (1.01–2.08)	0.042	1.38 (0.33–5.7)	0.657
Contact	28 (5.1)	516 (94.9)	1.96 (1.3–2.94)	0.001	1.65 (1.09–2.51)	0.018^*∗*^
Facility-based survey	2 (2.9)	67 (97.1)	1.07 (0.26–4.42)	0.920		
Follow-up	35 (18.8)	151 (81.2)	8.35 (5.62–12.4)	<0.005	1.32 (0.91–1.90)	0.145

## Data Availability

The data used to support the findings of this study are included within the article.

## References

[1] Rothan H. A., Byrareddy S. N. (2020). The epidemiology and pathogenesis of coronavirus disease (COVID-19) outbreak. *Journal of Autoimmunity*.

[2] Dhama K., Khan S., Tiwari R. (2020). Coronavirus disease 2019–COVID-19. *Clinical Microbiology Reviews*.

[3] Guo Y. R., Cao Q. D., Hong Z. S. (2020). The origin, transmission and clinical therapies on coronavirus disease 2019 (COVID-19) outbreak—an update on the status. *Military Medical Research*.

[4] Wang C., Horby P. W., Hayden F. G., Gao G. F. (2020). A novel coronavirus outbreak of global health concern. *The Lancet*.

[5] Valencia D. N. (2020). Brief Review on COVID-19: The 2020 Pandemic Caused by SARS-CoV-2. *Cureus*.

[6] Kakodkar P., Kaka N., Baig M. (2020). A Comprehensive Literature Review on the Clinical Presentation, and Management of the Pandemic Coronavirus Disease 2019 (COVID-19). *Cureus*.

[7] WHO (2020). *Statement on the Second Meeting of the International Health Regulations (2005) Emergency Committee Regarding the Outbreak of Novel Coronavirus (2019-nCoV)*.

[8] John Hopkins Coronavirus Resource Center (2021). *Mortality Analyses*.

[9] WHO (2020). *COVID-19 COVID-19. WHO J Covid 19, Reg Situational Updat Africa*.

[10] World Health Organization (WHO) (2021). *COVID-19 Region Situtational Update for WHO African Region*.

[11] Ozili P. (2020). COVID-19 in Africa: socio-economic impact, policy response and opportunities. *International Journal of Sociology and Social Policy*.

[12] Lone S. A., Ahmad A. (2020). COVID-19 pandemic–an African perspective. *Emerging Microbes and Infections*.

[13] Achoki T., Alam U., Were L. (2020). *COVID-19 Pandemic in the African Continent: medRxiv*.

[14] WHO (2020). *WHO Coronavirus Disease (COVID-19) Dashboard*.

[15] World Health Organization (2020). *WHO Coronavirus Disease (COVID-19) Dashboard*.

[16] Shrestha G. S. (2020). COVID-19 pandemic: shortage of personal protective equipment, use of improvised surrogates, and the safety of health care workers. *Journal of Nepal Health Research Council*.

[17] Chaib F. (2020). *Shortage of Personal Protective Equipment Endangering Health Workers Worldwide*.

[18] Rowan N. J., Laffey J. G. (2020). Challenges and solutions for addressing critical shortage of supply chain for personal and protective equipment (PPE) arising from Coronavirus disease (COVID19) pandemic—case study from the Republic of Ireland. *Science of the Total Environment*.

[19] Gilbert M., Pullano G., Pinotti F. (2020). Preparedness and vulnerability of African countries against importations of COVID-19: a modelling study. *Lancet*.

[20] Dzinamarira T., Dzobo M., Chitungo I. (2020). COVID-19: a perspective on Africa’s capacity and response. *Journal of Medical Virology*.

[21] Nuwagira E., Muzoora C. (2020). Is sub-saharan Africa prepared for COVID-19?. *Tropical Medicine and Health*.

[22] MOH (2020). First case of COVID-19 confirmed in Ethiopia.

[23] World Health Organization (2020). Global COVID-19 clinical platform Novel Coronavirus (COVID-19)—rapid version.

[24] Shirato K., Nao N., Katano H. (2020). Development of genetic diagnostic methods for detection for novel coronavirus 2019 (nCoV-2019) in Japan. *Japanese Journal of Infectious Diseases*.

[25] Li J., Qi S., Zhang C. (2013). A two-tube multiplex reverse transcription PCR assay for simultaneous detection of sixteen human respiratory virus types/subtypes. *BioMed Research International*.

[26] Zhang N., Wang L., Deng X. (2020). Recent advances in the detection of respiratory virus infection in humans. *Journal of Medical Virology*.

[27] Harkess G. (2019). Real-time fluorescent RT-PCR kit for detecting 2019-nCoV. *New Scientist*.

[28] Gebretsadik D., Ahmed N., Kebede E., Gebremicheal S., Belete M. A., Adane M. (2021). Knowledge, attitude, practice towards COVID-19 pandemic and its prevalence among hospital visitors at Ataye district hospital, Northeast Ethiopia. *PLoS One*.

[29] Gudbjartsson D. F., Helgason A., Jonsson H. (2020). Spread of SARS-CoV-2 in the Icelandic population. *New England Journal of Medicine*.

[30] Snoeck C., Vaillant M., Abdelrahman T. (2020). *Prevalence of SARS-CoV-2 Infection in the Luxembourgish Population: The CON-VINCE Study*.

[31] Maver Vodičar P., Oštrbenk Valenčak A., Zupan B. (2020). Low prevalence of active COVID-19 in Slovenia: a nationwide population study of a probability-based sample. *Clinical Microbiology and Infection*.

[32] Khan M., Khan H., Khan S., Nawaz M. (2020). Epidemiological and clinical characteristics of coronavirus disease (COVID-19) cases at a screening clinic during the early outbreak period: a single-centre study. *Journal of Medical Microbiology*.

[33] Elhakim M., Banoita Tourab S., Zouiten A. (2020). COVID-19 pandemic in Djibouti: epidemiology and the response strategy followed to contain the virus during the first two months, 17 March to 16 may 2020. *PLoS One*.

[34] World Health Organization (2020). *Coronavirus Disease 2019 Situation Report*.

[35] Pan A., Liu L., Wang C. (2020). Association of public health interventions with the epidemiology of the COVID-19 outbreak in Wuhan, China. *JAMA—Journal of American Medical Association*.

[36] Haftom M., Petrucka P., Gemechu K. (2020). Knowledge, attitudes, and practices towards covid-19 pandemic among quarantined adults in Tigrai region, Ethiopia. *Infection and Drug Resistance*.

[37] Li S., Feng B., Liao W., Pan W. (2020). Internet use, risk awareness, and demographic characteristics associated with engagement in preventive behaviors and testing: cross-sectional survey on COVID-19 in the United States. *Journal of Medical Internet Research*.

[38] Clements J. M. (2020). Knowledge and behaviors toward COVID-19 among us residents during the early days of the pandemic: cross-sectional online questionnaire. *Journal of Medical Internet Research*.

[39] Huynh G., Nguyen M. Q., Tran T. T. (2020). Knowledge, attitude, and practices regarding COVID-19 among chronic illness patients at outpatient departments in Ho chi minh city, Vietnam. *Risk Management and Healthcare Policy*.

[40] Ngwewondo A., Nkengazong L., Ambe L. A. (2020). Knowledge, attitudes, practices of/towards COVID 19 preventive measures and symptoms: a cross-sectional study during the exponential rise of the outbreak in Cameroon. *PLoS Neglected Tropical Diseases*.

[41] Akalu Y., Ayelign B., Molla M. D. (2020). Knowledge, attitude and practice towards covid-19 among chronic disease patients at addis zemen hospital, Northwest Ethiopia. *Infection and Drug Resistance*.

[42] Zhao C., Wong L., Zhu Q., Yang H. (2019). Prevalence and correlates of chronic diseases in an elderly population: a community-based survey in Haikou. *PLoS One*.

[43] Kuri-Morales P., Emberson J., Alegre-Díaz J. (2009). The prevalence of chronic diseases and major disease risk factors at different ages among 150 000 men and women living in Mexico City: cross-sectional analyses of a prospective study. *BMC Public Health*.

[44] Atella V., Piano Mortari A., Kopinska J. (2019). Trends in age-related disease burden and healthcare utilization. *Aging Cell*.

[45] Zhou F., Yu T., Du R. (2020). Clinical course and risk factors for mortality of adult inpatients with COVID-19 in Wuhan , China: a retrospective cohort study. *Lancet [Internet]*.

[46] Wu C., Chen X., Cai Y. (2020). Risk factors associated with acute respiratory distress syndrome and death in patients with coronavirus disease 2019 pneumonia in Wuhan, China. *JAMA Internal Medicine*.

[47] Dong Y., Dong Y., Mo X. (2020). Epidemiology of COVID-19 among children in China. *Pediatrics*.

[48] Wu C., Chen X., Cai Y. (2020). Risk factors associated with acute respiratory distress syndrome and death in patients with coronavirus disease 2019 pneumonia in Wuhan, China. *JAMA Internal Medicine*.

[49] Chinazzi M., Davis J. T., Ajelli M. (2020). The effect of travel restrictions on the spread of the 2019 novel coronavirus (COVID-19) outbreak. *Science*.

